# Free Senior High School Lunch Contributes to Dietary Quality of Nonresidential Students in Ghana

**DOI:** 10.1177/0379572120970828

**Published:** 2020-11-15

**Authors:** Abdul-Razak Abizari, Zakari Ali, Seidu Alhassan Abdulai, Fauzia Issah, Nana Adwoa Frimpomaa

**Affiliations:** 1 128415 University for Development Studies, Tamale, Ghana; 2 47969 MRC Unit, The Gambia at the London School of Hygiene and Tropical Medicine, Banjul, The Gambia

**Keywords:** school feeding, adolescents, dietary diversity, senior high schools, Ghana

## Abstract

**Background:**

School feeding offers an excellent opportunity for targeted intervention to students not only as means for improving educational outcomes but also enhancing nutritional outcomes. The Government of Ghana introduced the free lunch feeding policy for nonresidential students in senior high schools (SHS) in 2018.

**Objective:**

We assessed unintended benefits of the free lunch program to dietary improvement.

**Methods:**

This was an analytical cross-sectional study among 403 (202 beneficiary and 201 non-beneficiary) students in SHS. The Food and Agriculture Organization’s standard procedure for measuring dietary diversity score (DDS) was followed. A 3-day dietary recall was used to assess school day DDS, while a 24-hour recall was used to assess weekend DDS of students. Differences in DDS and food group consumption were determined using student *t* test and χ^2^ test, respectively.

**Results:**

Nearly all (98.5%) beneficiary students consumed the free school lunch and 7 (70%) in 10 of them consumed it on all school days. While the students did not differ in their weekend meal DDS (6.3 ± 1.4 vs 6.5 ± 1.4, *P* = .39), beneficiaries of the school lunch had higher lunch DDS (7.5 ± 0.5 vs 6.5 ± 1.4, *P* < .001) and whole day DDS (11.5 ± 1 vs 9.3 ± 2.0, *P* < .001) compared to non-beneficiary students on school days. Even though the school lunch increased food group intake, vitamin-A rich vegetables and tubers, fruits, flesh and organ meats, and dairy products were hardly provided as components of school lunch.

**Conclusion:**

Provision of free school lunch meal to nonresidential students in SHSs in Ghana could contribute to improved diet quality.

## Introduction

Nutritional challenges in developing countries are high and continue to impair health, quality of life, and survival.^1^ Nutrition interventions have principally targeted the prevention of malnutrition during periods of rapid growth such as during childhood and adolescence.^2^ Once final height is attained at adolescence, the consequence of stunting becomes uncorrectable. The growth spurt of adolescence has been seen as a period of potential interest for catching up growth deficit of childhood.^3^ Optimal nutrition is therefore an important consideration at this critical stage of life. The provision of hot school meals offers an excellent opportunity for targeted intervention of adolescents and serves as both a means for enhancing nutrition and improving attendance and educational outcomes.^4^

Diversity in the food adolescents eat is necessary in improving quality and meeting the requirements for energy and nutrients. Inadequate dietary diversity is particularly problematic among poor populations in developing countries. Therefore, increasing the diversity of food available for consumption could reduce both undernutrition and overnutrition.^5,6^

School feeding in Ghana has mostly operated at the primary school level with expected outcomes including poverty reduction, increased school enrolment, improved food security, and improved nutritional status. The program has recorded marked increase in school enrolment, reduced gender gap between boys and girls, and improved nutritional status in the beneficiary schools.^7,8^

In the light of these successes of school feeding in the primary schools, the government of Ghana introduced a policy on free school feeding in senior high schools (SHS) in September 2017. The aims were to increase enrolment and potentially improve nutritional status of students in SHSs. The policy includes 3 square meals for residential students and a hot lunch for nonresidential students. The policy has been implemented in a progressive approach starting with students in the first year of SHS admitted in the 2017/2018 academic year. As beneficiary nonresidential students are expected to take other meals at home, the free lunch meal is essentially expected to be an addition to the overall dietary intake in the day. However, it is unknown whether the free lunch makes significant improvements to the dietary quality of beneficiary nonresidential students. To our knowledge, the contribution of the free school lunch to dietary improvement of students has not been assessed since the inception of the policy. Such data could be useful in informing policy makers on the progressive scale-up of the program. Therefore, the present study aimed to assess the contribution of the free school lunch to dietary improvement as measured through dietary diversity of students who attend SHSs in Northern Ghana.

## Methods

### Study Design and Area

This study was an analytical cross-sectional study conducted in the Tamale Metropolis in May and June 2018. Tamale is the capital of the Northern Region of Ghana and is the fourth largest city in Ghana. It is the principal center of education in the region. As of 2018, the Metropolis had 14 SHSs.^9^ The study was conducted in 3 SHSs: Vitting SHS (Vitting), Kalpohini SHS (Kalpohini), and Northern School of Business (Nobisco). As most schools in the Metropolis operate mainly residential student status, we purposively selected schools with a good balance of residential and nonresidential student numbers.

### Study Population and Sampling

The target population was nonresidential students in SHSs in the Tamale Metropolis. The free school lunch policy started with first year students admitted in the 2017/2018 academic year. Therefore, as of the time of this study, there was only 1-year group (SHS 1) benefiting from the free school lunch. Nonresidential students in the SHS 2 served as comparison group. The study sample consisted of beneficiaries (SHS 1 students) and non-beneficiaries (SHS 2 students) in the schools. A total of 403 students (202 beneficiary and 201 non-beneficiary) were selected to participate in the study. The number of students enrolled per school was determined through probability proportionate to size of school. Therefore, more students were sampled from schools with larger populations. A list of nonresidential students was prepared from class registers for each school and used to select students through simple random sampling using Excel-generated random numbers. Selected students were identified in their respective classes and followed for questionnaire administration.

### Data Collection

Pretested semi-structured questionnaires were used to elicit participant information on sociodemographic characteristics, household wealth, hunger indicators, and student perceptions about the free school lunch.

### Assessment of Dietary Diversity and Hunger

A qualitative 24-hour and 3-day dietary recall of foods consumed by the respondents was used. The 24-hour recall was used for weekend meals, while the 3-day recall was used for school day meals. As school lunch is not served to nonresidential students during weekends, the weekend dietary assessment provided data on usual intake outside the influence of the school lunch. Dietary assessment was therefore conducted on 2 different days. The first dietary assessment was on either Thursdays or Fridays, where 3-day recalls were made to allow for recall of foods taken from Monday onward. The second dietary assessment took place the following Monday, where participants made a 24-hour recall of foods taken during the previous day (weekend/nonschool day). The Food and Agriculture Organization’s standard approach to assessing dietary diversity based on 14 food groups was followed.^10^ The dietary diversity score (DDS) was calculated from a simple count of the number of food groups a participant consumed over a specified recall period. Therefore, the DDS ranged from 0 when none of the food groups were consumed to 14 when all food groups were consumed. The food groups used for the calculation of the DDS were (1) cereals; (2) white roots and tubers; (3) vitamin A-rich vegetables and tubers; (4) dark green leafy vegetables; (5) other vegetables; (6) vitamin A-rich fruits; (7) other fruits; (8) organ meat; (9) flesh meat,; (10) eggs; (11) fish and sea foods; (12) legumes, nuts, and seeds; (13) milk and milk products; and (14) fats and oils. For the free lunch beneficiaries, in addition to the dietary recall, school kitchen menus were examined. School meal menus were obtained from school kitchens. The data reported for 3-day lunch food groups and 24-hour lunch DDS for beneficiary students represent the free lunch. Other foods taken at lunch are included in the 3 whole day recall data. Questionnaires were interviewer-administered by trained final year undergraduate nutrition students and took place at the schools during break time.

Household hunger was assessed and classified according to the Household Hunger Scale.^11^ The tool consists of 3 questions and 3 sets of frequencies (never, rarely or sometimes, and often) which pertains to household experience of food insufficiencies. It allows classification of households into 3 main hunger categories (little to no household hunger; moderate household hunger; and severe household hunger).^11^ Household responses to the 3 questions were scored as follows: never = 0, rarely or sometimes = 1, and often = 2. The total scores ranged from 0 to 6; households scoring 0 to 2 were classified as having “little to no hunger”; those with scores 2 to 3 had “moderate household hunger”; and scores 4 to 6 were classified as “severe household hunger.”^11^

### Data Analysis

Data were entered, cleaned, and analyzed using SPSS version 21. Categorical data have been presented as frequencies and percentages, while continuous data are reported as means and SD. Unpaired student *t* test was used to test differences in DDSs (continuous variable) among beneficiary and non-beneficiary students. Comparison of food group intake was made using χ^2^ test.

### Research Ethics and Patient Consent

The students or their parents (where students were younger than 18 years) signed an informed consent before participating in the study. Participation in the study was voluntary, and where parents consented on behalf of students, we also obtained student assent to participate. The study protocol was also approved by the Scientific Review Committee of the School of Allied Health Sciences, University for Development Studies, Ghana.

## Results

### Background Characteristics of Beneficiary and Non-Beneficiary Students

Six (59.8%) in 10 of sampled students were male, but there were important sex differences between beneficiary and non-beneficiary students. More than half (52%) of the students were aged 12 to 17 years with non-beneficiaries being a little older (*P* < .001). There was no statistical evidence of a difference in participant’s religion and mode of transport to school. A larger proportion of the students belonged to the Dagomba ethnic group (*P* = .009) and had their fathers as household heads (*P* = .02). There were similar occupations of household heads (*P* = .48), household hunger classification (*P* = .95), source of household food (*P* = .49), and household wealth classification (*P* = .05; Table 1).

**Table 1.** Background Characteristics of Beneficiary and Non-Beneficiary Students.

Characteristic	Beneficiary (n = 202) n (%)	Non-beneficiary (n = 201) n (%)	Total n (%)	*P* value
Sex				<.001
Male	146 (72.3)	95 (47.3)	241 (59.8)	
Female	56 (27.7)	106 (52.7)	162 (40.2)	
Age (years)				
12-17	138 (68.3)	70 (34.8)	208 (51.6)	<.001
≥18	64 (31.7)	131 (65.2)	195 (48.4)	
Religion				.06
Islam	158 (78.2)	172 (85.6)	330 (81.9)	
Christianity	44 (21.8)	29 (14.4)	73 (18.1)	
Ethnicity				.01
Dagomba	130 (64.4)	159 (79.1)	289 (71.7)	
Mamprusi	7 (3.5)	5 (2.5)	12 (3.0)	
Gonja	12 (5.9)	10 (5.0)	22 (5.5)	
Akan	12 (5.9)	10 (5.0)	22 (5.5)	
Others	41 (20.3)	17 (8.5)	58 (14.4)	
Means to school				.30
Foot	122 (60.4)	138 (68.7)	260 (64.5)	
Bicycle	46 (22.8)	32 (15.9)	78 (19.4)	
Motorcycle	17 (8.4)	15 (7.5)	32 (7.9)	
Car	17 (8.4)	16 (8.0)	33 (8.2)	
Household head				.02
Father	138 (68.3)	110 (54.7)	248 (61.5)	
Mother	18 (8.9)	22 (10.9)	40 (9.9)	
Grandfather	15 (7.4)	12 (6.0)	27 (6.7)	
Grandmother	8 (4.0)	9 (4.5)	17 (4.2)	
Uncle	17 (8.4)	39 (19.4)	56 (13.9)	
Others	6 (3.0)	9 (4.5)	15 (3.7)	
Occupation of household head				.48
Trader	49 (24.3)	61 (30.3)	110 (27.3)	
Agricultural worker	82 (40.6)	64 (31.8)	146 (36.2)	
Office worker	14 (6.9)	12 (6.0)	26 (6.5)	
Service worker	7 (3.5)	5 (2.5)	12 (3.0)	
Education/research	16 (7.9)	18 (9.0)	34 (8.4)	
Health worker	3 (1.5)	2 (1.0)	5 (1.2)	
Unemployed	15 (7.5)	24 (11.9)	39 (9.7)	
Others	16 (7.9)	15 (7.5)	31 (7.7)	
Household source of foods				.49
Own production	97 (48.0)	79 (39.3)	176 (43.7)	
Purchase	94 (46.5)	109 (54.2)	203 (50.4)	
Food aid/gift	6 (3.0)	3 (1.5)	9 (2.2)	
Borrowing	5 (2.5)	10 (5.0)	15 (3.7)	
Household hunger category				.95
Severe	5 (2.5)	5 (2.5)	10 (2.5)	
Moderate	45 (22.3)	42 (20.9)	87 (21.6)	
No hunger	152 (75.2)	154 (76.6)	306 (75.9)	.05
Household wealth category				
High	114 (56.4)	94 (46.8)	208 (51.6)	
Low	88 (43.6)	107 (53.2)	195 (48.4)	

### Dietary Practices and Perceptions of Free School Lunch

Majority of the sampled students reported to school with pocket money (66.3% vs 64.2%; *P* = .65; for beneficiary and non-beneficiary students, respectively). Reports about buying food at school were higher (*P* < .001) among non-beneficiary students (54.5% vs 82.4% for beneficiary and non-beneficiary students, respectively; data not shown for non-beneficiary students). In addition to the school lunch, 72% of beneficiary students had additional meals for lunch. For 48% of the beneficiaries, additional meals were taken because they still felt hungry. For other beneficiaries (22%), additional meals were taken because food was available at home on return from school (Table 2).

**Table 2.** Dietary Practices and Perceptions of Free Lunch Beneficiaries.

Practice/perception	Frequency (%)
Do you come to school with pocket money	
Yes	134 (66.3)
What do you use pocket money for?	
Buy food	73 (54.5)
By water	38 (28.4)
Buy fruits	3 (2.2)
Buy snacks	18 (13.4)
Others	2 (1.5)
Use of pocket money before free lunch	
Buy food	100 (74.6)
Buy water	23 (17.2)
Buy fruits	1 (0.7)
Buy snacks	8 (6.0)
Other	2 (1.5)
Do you take lunch at home?	
Yes	146 (72.3)
Why do you take lunch at home?	
I do not like the school lunch	5 (2.5)
Still feel hungry after eating the school lunch	97 (48.0)
There is food when I get to the house	44 (21.8)
Did you take school lunch yesterday?	
Yes	199 (98.5)
Frequency of school lunch in a week	
All 5 days	141 (69.8)
Others	62 (30.2)
How do you feel about the free lunch?	
It alleviates hunger	105 (52.0)
It is nutritious	15 (7.4)
It is a balance diet	64 (31.7)
Others	18 (8.8)
Diet more diversified now?^a^	
Yes	194 (96.0)
Reasons for diet more diversified	
Because I eat from all food groups	12 (5.9)
Because I eat from more than 5 food groups	18 (8.9)
Because I eat 3 times a day	113 (56.4)
Because I include fruit and vegetables in my diet	51 (25.2)

^a^ Diversified diet was defined as a diet with more food groups.

Nearly all (98.5%) the beneficiary students had taken the free school lunch the day preceding the survey, and 7 (70%) in 10 of them take it on all school days. More than half (52%) of them felt the free school lunch alleviates hunger, and close to one-third (31.7%) thought it was a balanced meal and a few others (7.4%) thought it was nutritious. Almost all beneficiary students (96.0%) felt the introduction of the free lunch has improved the diversity of their diet; because the number of times they eat during a school day has increased (56.4%), they now consume fruits and vegetables (25.2%) and eat from more food groups (9.0%; Table 2).

### Comparison of Weekend Food Consumption of Beneficiary and Non-Beneficiary Students

**Figure 1.**
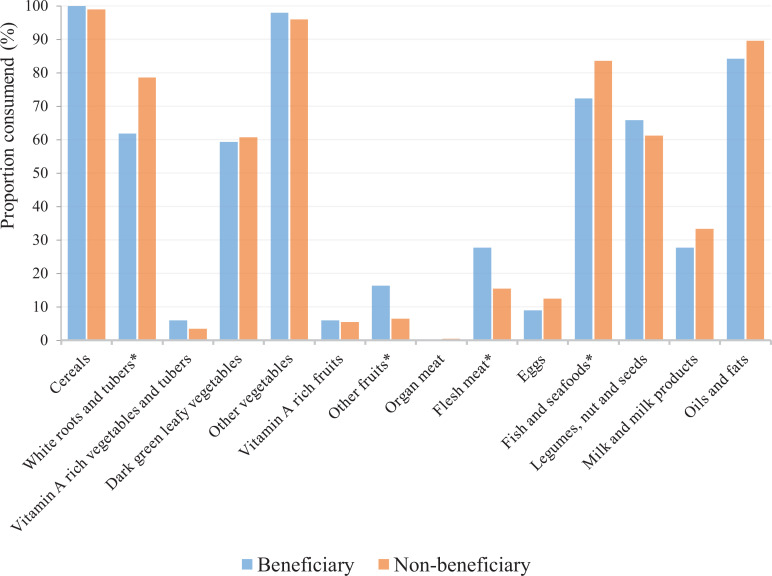
Comparison of weekend food groups among free lunch and nonschool lunch beneficiaries. * indicate a statistically significant difference in consumption between beneficiary and non-beneficiary students.

Of the 14 food groups, participants differed in only 4 food groups during the weekend. These food groups were white roots and tubers, other fruits, flesh meats, and fish and seafood. While non-beneficiary students consumed more of white roots and tubers (*P* = .001) and fish and seafood (*P* = .006), beneficiary students mostly consumed other fruits (*P* = .002) and flesh meats (*P* = .003; Figure 1).

### Comparison of Lunch and Whole Day Food Groups Among Beneficiary and Non-Beneficiary Students (3-Day Recall)

Details of foods served as school lunch in the schools is provided in Table 3. The menu for the 5 days in all schools are foods mainly made from cereals, legumes, and white roots and tubers. The meal is served in such a way that if cereal is served today, legumes or white roots will be served tomorrow. Also, all meals in the 5 days are prepared using oil, fish, spices, and other vegetables. It is only Vitting SHS that includes Amaranth leaves which is a dark green leafy vegetable in their menu and it is served only on Fridays. Nine of the 16 food groups used in the interview were served as school lunch across almost all schools.

**Figure 2.**
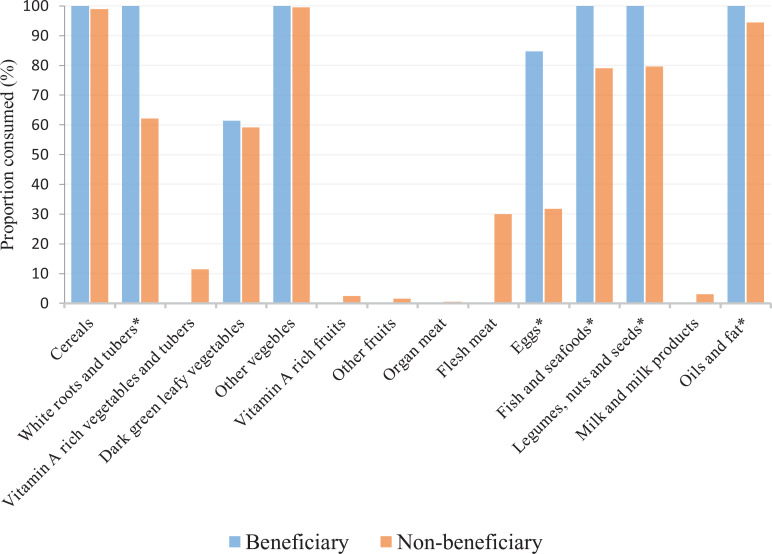
Comparison of 3-day lunch food groups among beneficiary and non-beneficiary students. * indicate a statistically significant difference in consumption between beneficiary and non-beneficiary students.

Even though the school lunch increased food group intake, vitamin A-rich vegetables and tubers; fruits, flesh and organ meats; and dairy products were hardly provided as school lunch. For example, less than 5% of all sampled students consumed from vitamin A-rich fruits, other fruits, organ meats, and milk and milk products in the 3-day recall of lunch meals. There was no evidence of a difference in consumption of cereals and other vegetables at lunch. Beneficiary students, however, had a higher reported frequency of consumption of white roots and tubers, eggs, fish and seafood, legumes and nuts, and fats and oils taken as school lunch (*P* < .001; Figure 2).

**Table 3.** Menus of Free Lunch Among Participating Schools.^a^

Day	Name of school	Name of dish	Main ingredients
Monday	Kalpohin	Bean Stew and Yam Slices	Beans, yam, vegetable oil, pepper, salt, saltpeter, tomatoes, onion
	Nobisco	*Gari* and Beans	*Gari*, beans, palm oil, anchovies, salt, onion, tomatoes, saltpeter
	Vitting	*Gari* and Beans	Beans, *Gari*, fish, palm oil, onion, tomatoes, pepper, saltpeter
Tuesday	Kalpohin	*Jollof* with Egg	Rice, eggs, bouillon cubes, spices, anchovies, tomatoes, onions, oil
	Nobisco	Plain Rice with Amaranth Sauce	Amaranth leaves, *agushie* (white melon seeds), salted fish, onion, pepper, rice, canned fish, bouillon cubes, tomatoes, vegetable oil
	Vitting	*Jollof* with Egg	Rice, eggs, vegetable oil, canned tomatoes, onion, pepper, bouillon cubes, anchovies, salt
Wednesday	Kalpohin	*Gari* and Beans	Beans, *Gari*, salt, pepper, saltpeter, anchovies, palm oil, onion
	Nobisco	Rice and Beans (*waakye*)	Rice, beans, vegetable oil, keta boys, bouillon cubes, spices, onion, salt, pepper
	Vitting	*Gari* and Beans	Beans, *Gari*, fish, palm oil, onion, tomatoes, pepper, saltpeter
Thursday	Kalpohin	Rice and Beans *Jollof*	Rice, beans, tomatoes, salt, onion, anchovies, vegetable oil, saltpeter
	Nobisco	Rice Jollof	Rice, onion, vegetable oil, canned fish, bouillon cubes, spices
	Vitting	Rice and Beans *Jollof*	Rice, beans, vegetable oil, canned tomatoes, onion, bouillon cubes, salt
Friday	Kalpohin	Crushed maize and Beans *Jollof*	Maize, beans, pepper, salt, vegetable oil, anchovies, saltpeter
	Nobisco	*Gari* and Beans	*Gari*, beans, palm oil, anchovies, salt, onion, salted fish, tomatoes, pepper
	Vitting	Plain Rice with Amaranth Sauce	Rice, pepper, palm nut, Amaranth leaves, tomatoes, salt, onions, bouillon cubes, anchovies

^a^ Jollof: A meal of rice made with oil and tomato sauce. Gari: A local preparation of roasted cassava flour.

**Figure 3.**
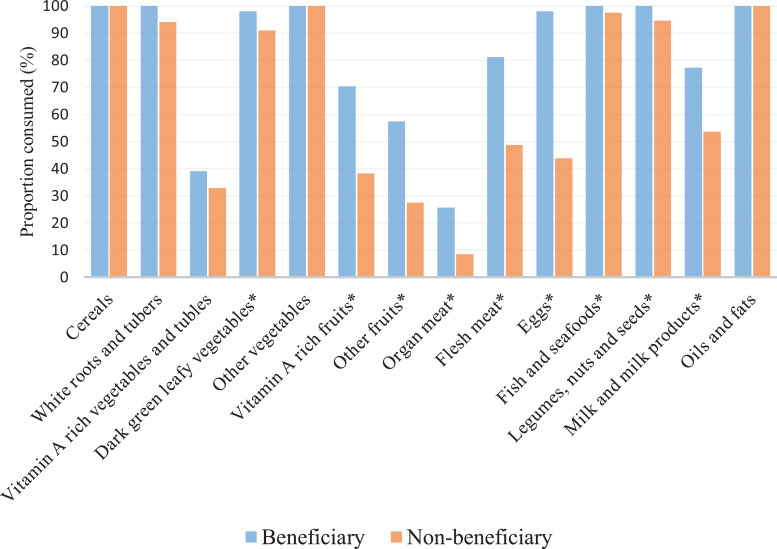
Comparison of whole day food groups among beneficiary and non-beneficiary students (3-day recall) * indicate a statistically significant difference in consumption between beneficiary and non-beneficiary students.

In a 3-day recall of whole day meals, more than 90% of the students consumed cereals, white roots and tubers, dark green leafy vegetables, other vegetables, fish and seafood, legumes and nuts, and fats and oils. A statistically significant higher proportion of beneficiary students consumed from 8 food groups than non-beneficiary students in the following food groups: dark green leafy vegetables, vitamin-A rich fruits and vegetables, other fruits, organ meats, eggs, fish and seafood, legumes and nuts, and milk and milk products (Figure 3).

### Comparison of Mean DDSs Among Beneficiary and Non-Beneficiary Students

There was no evidence (*P* = .39) that participants differed in mean DDS of weekend meals (6.3 vs 6.5). However, there was strong evidence of a 1-unit increase in DDS of beneficiary students at lunch (7.5 vs 6.5, *P* < .001) and 2-unit increase in whole day DDS of beneficiary students (DDS = 11.5) compared to non-beneficiary students (DDS = 9.3; *P* < .001) on weekdays (Table 4).

**Table 4.** Comparison of Mean DDSs Among Beneficiary and Non-Beneficiary Students.

	Beneficiary (mean ± SD)	Non-beneficiary (mean ± SD)	Total (mean ± SD)	*P* value
Weekend dietary diversity (24-hour recall)				
Whole day DDS	6.3 ± 1.4	6.5 ± 1.4	6.4 ± 1.4	.387
Weekday dietary diversity (3-day recall)				
Lunch DDS	7.5 ± 0.5	6.5 ± 1.4	7.0 ± 1.1	<.001
Whole day DDS	11.5 ± 1.5	9.3 ± 2.0	10.4 ± 2.0	<.001

Abbreviation: DDS, dietary diversity score.

## Discussion

We sought to assess the contribution of free school lunch to dietary improvement of students who attend SHSs in Northern Ghana. The main findings are that most beneficiary students took the free school lunch on all school days. Dietary diversity scores did not differ during the weekends among beneficiary and non-beneficiary students. The provision of free school lunch, however, led to a 1-unit increase in DDSs at lunch among beneficiary students compared to non-beneficiaries. It also led to 2-unit increase in DDSs of whole day meals of beneficiary students compared to non-beneficiaries. Foods from vitamin A-rich vegetables and tubers, fruits, flesh and organ meats, and dairy products groups were hardly provided as components of school lunch.

The higher number of students who reported taking the school lunch on all school days could indicate high acceptance and uptake of the free lunch policy. As household hunger indicators were similar and dietary diversity of participants did not differ during weekends, the higher DDSs among beneficiary students during school days could be attributed to the supplementary effects of foods taken as free lunch. Higher dietary diversity in food consumption may indicate adequate nutrients intake.^12^ An earlier study in the same region also found high energy and nutrient intake among school feeding beneficiaries in primary schools.^13^ The free school lunch may have improved dietary diversity of students by increasing their access to different food groups.^14^ In other studies, the provision of school lunch increased dietary diversity, school enrolment, attendance, and student concentration in class.^15^ A national school lunch program has been found to be a major policy effort to alleviate the problems associated with hunger and a lack of adequate nutrition among low-income children.^16^

As beneficiary students still came to school with pocket money and a majority spent it on food and also had some foods at home after school, this could explain the 2-unit increase in DDSs of whole day meals even though they had one food group increase at only lunch meals. Foods bought at school in addition to the school lunch therefore led to additional increase in the number of food groups taken in the day. With targeted education, pocket money could be used to buy foods that are often not served as part of the school lunch such as fruits, meats, vitamin A-rich foods, and dairy products. This could be implemented through a verbal prompt intervention which has been shown to be associated with a higher likelihood that students will spend their pocket money on fruits.^17^

Beneficiaries of the free school lunch had high intake of white roots and tubers, eggs, fish, and seafood which are main components of meals provided as school lunch and could explain the high intake of these food groups at lunch. Non-beneficiary students consumed more flesh meats from lunch meals which could be due to foods bought from school during lunch time which usually include components of flesh meat or fish. The reason beneficiary students had higher whole day consumption of flesh meats in the whole day despite low intakes from school lunch could be due to additional food purchase outside of lunch, potentially at closing time when they would have consumed lunch hours ago.

Foods from vitamin A-rich vegetables and tubers, fruits, flesh and organ meats, and dairy products groups were hardly provided as components of school lunch. Similar results were reported earlier among primary schools where eggs, dairy, and fruits were never served as part of a school feeding program in the Volta region of Ghana.^18^ While meats, fruits, and foods rich in vitamin A such as carrots could simply be served as part of the common meals on the school lunch menus, dairy products are hardly part of typical Ghanaian lunch dishes. Foods such as *Waagashie*, a local cheese, normally fried and served with a variety of foods could be included in the school lunch menu to enable intake of dairy products. The poor intake of dairy products could be an opportunity for the dairy industry to explore novel ways to include dairy in the free lunch program to promote intake and improve nutrition. There are relatively fewer SHSs and food storage facilities are more improved at this level, so it could be a suitable and sustainable stage to implement a targeted national dairy consumption improvement program as a pilot. A recent study explored the potential to increase the protein content of school lunch served in basic schools and found locally produced soy flour preparations to be cost effective compared to animal source alternatives such as ground beef and mackerel and could also be included in the school lunch menu at the SHS level.^19^

The findings of this study could inform policy decisions of the government of Ghana on the free school lunch policy for nonresidential students in SHSs to ensure a more diverse and nutritious meal provision in schools. However, the findings need to be interpreted keeping some study limitations in mind. There could be recall problems with the dietary data reported in this study. However, we do not expect this to have much impact on the validity of the results because similar effects would have been observed in both groups. The strength of this study is the measurement of weekend dietary intake which served as baseline to understand the dietary intake of both groups outside the influence of the school lunch.

## Conclusion

Provision of free school lunch meal to nonresidential senior high students in Ghana could contribute to improved diet quality.
